# Institutional gatekeeping of food and beverage marketing in schools and youth sport

**DOI:** 10.3389/fnut.2026.1953630

**Published:** 2026-10-19

**Authors:** Lu Xing, Xueqi Wu

**Affiliations:** 1 School of Preschool Education, Changsha Normal University, Changsha, China; 2 School of Education, Faculty of Social Sciences & Leisure Management, Taylor’s University, Subang Jaya, Malaysia; 3 Taylor’s Culinary Institute, Faculty of Social Sciences & Leisure Management, Taylor’s University, Subang Jaya, Malaysia

**Keywords:** children, food marketing, food policy, institutional gatekeeping, school food environment, sport sponsorship

## Abstract

Food and beverage marketing to children is usually framed as communication between firms and individual consumers. In schools and organized youth sport, however, exposure depends on institutional decisions about procurement, fundraising, sponsorship, branded programs, facility advertising, equipment, awards, and food-service contracts. This Mini Review synthesizes evidence from these settings through an institutional-gatekeeping lens and asks how commercial access is granted, which organizational constraints sustain it, and how directly the evidence connects exposure with nutrition outcomes. School studies show that formal nutrition standards can coexist with fundraising, incentive programs, and corporate signage alongside differences in policy coverage and implementation capacity. Youth-sport studies likewise identify pervasive branded infrastructure, sponsorship, and service contracts, often involving products of nutritional concern. Across both settings, direct evidence is concentrated on marketing presence, product healthfulness, brand recall, and attitudes; the next step is to link institution-specific exposure with dietary intake and longer-term health outcomes. Policy options arising from this synthesis include rules governing commercial access and brand-level promotion, contract transparency and monitoring, and feasible replacement resources, with their effects on exposure and participation requiring evaluation. Research priorities include lower-resource systems, implementation outcomes, and links between institutional exposure and dietary behavior.

## 1 Introduction

Commercial food environments shape what children notice, normalize, request, and select (1, 2). Research and regulation often emphasize messages directed at children. In schools and youth sport, boards, administrators, parent organizations, clubs, facility managers, and contractors also determine which firms can enter. Their decisions connect brands with trusted institutions and control access to organizational spaces, assets, and activities.

A school may prohibit a product’s sale while displaying its parent brand through fundraising; a sport facility may adopt nutrition guidance while retaining branded infrastructure or an exclusive service contract. Earlier reviews established heterogeneous school marketing and associations between unhealthy products and positive sporting meanings (1–3). Recent evidence on contracts, organizational capacity, and alternative sponsorship enables closer examination of these access arrangements.

Institutional gatekeeping is used here as an analytical label for organizational decisions that authorize, maintain, or constrain commercial access to children. It operates at the meso level, between firms and individuals, through spaces, activities, communication channels, and contractual rights. Fundraising, incentive programs, sponsorship, and food-service contracts illustrate these arrangements (4–6). Resource dependence is used descriptively for reliance on company-provided money, equipment, products, or services, with the degree of reliance shaped by available alternatives.

This Mini Review asks three questions. First, through which institution-controlled channels do food and beverage firms obtain access to schools and youth sport? Second, how do organizational resources, rules, and implementation capacity enable or constrain that access? Third, how directly does the evidence connect institution-mediated exposure with product healthfulness, child responses, food choice, intake, and health?

## 2 Scope and approach

This focused narrative review used database searches to build an evidence map, eligibility criteria to delimit scope, and purposive selection to assemble the narrative synthesis; it was not conducted as a systematic review. Web of Science Core Collection and Scopus were searched on 29 July 2026 for English-language articles and reviews published since 2000.

Searches combined food and beverage, marketing, child and adolescent, and school or youth-sport setting terms using database-adapted syntax. Offline year, document-type, and language checks and DOI/normalized-title deduplication yielded a pool of 893 records. Supplementary Table S1 provides the executed search strings, limits, and intermediate counts.

Eligible evidence concerned commercial food or non-alcoholic beverage marketing in schools or organized youth sport, including shared recreation facilities serving children. Institutional control could involve spaces, assets, digital platforms, contracts, procurement, fundraising, sponsorship, or events. Evidence could concern children, parents, personnel, or policymakers. Eligibility was independent of statistical significance, outcome direction, or explicit mechanism terminology. Simulated sponsorship experiments were classified separately from actual institutional exposure.

Exclusions covered household-only behavior; general media, retail, neighborhood, or elite-sport exposure without an institutional connection; unrelated adult research; availability or nutrition education without commercial marketing; alcohol-, tobacco-, gambling-, or breast-milk-substitute-only marketing; and noneligible publication types. Reviews and evaluations combining school rules with wider labeling and marketing policies provided contextual evidence.

Purposive selection considered relevant commercial arrangements and outcomes, extractable setting-specific findings, complementary designs and policy contexts, and null or contrasting results. Additional publications were identified through targeted re-examination of the original record pool and full-text checking. All 42 selected publications originated from that pool: 29 direct setting studies, two sport-sponsorship experiments, and 11 contextual publications. Supplementary Table S2 presents their characteristics, findings, and thematic contributions; Supplementary Table S2B provides the available candidate-map and citation records.

GPT-5 (OpenAI) performed publication selection, source checking, evidence extraction, and classification using these criteria. Lu Xing verified the selection and extracted information for all 42 publications against the original sources; Xueqi Wu cross-checked all 42 publications and their extraction records. Extraction covered design, setting, sample, commercial arrangements, institutional control, outcomes, findings, and thematic contributions. Mechanism information was classified as reported, inferred from arrangements, or not reported. Study-specific healthfulness classifications and denominators were retained; percentages from different contexts are not directly equivalent.

Findings were organized into four themes: commercial access and exposure; organizational rules, resources, and implementation; brand responses and sponsorship acceptability; and purchasing, dietary, and nutrition-related health outcomes. Publications could contribute to several themes. Shared samples were identified to avoid treating related reports as independent study populations.

No formal risk-of-bias assessment or certainty-of-evidence grading was undertaken. Figure 1 links the outcome domains and proposed policy actions.

**Figure 1:**
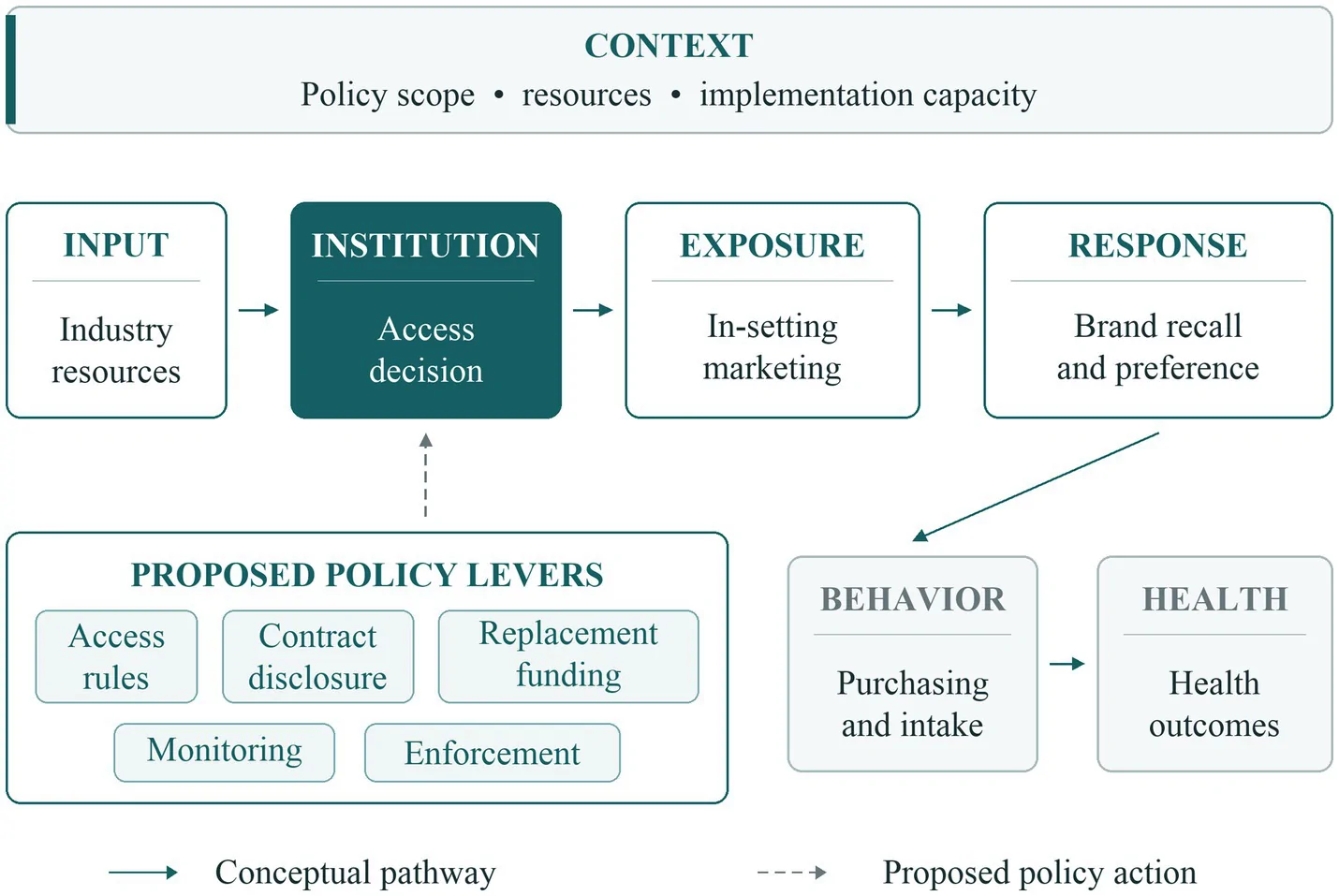
Conceptual pathway connecting institutional gatekeeping, marketing exposure, brand responses, and nutrition outcomes. Solid arrows represent the proposed conceptual pathway, not empirically established causal effects. Dashed arrows indicate proposed policy actions.

## 3 Schools: fragmented rules and multiple access channels

### 3.1 Commercial access and exposure

School commercialization extends beyond display advertising. Canadian evidence identified branded-food fundraising, on-site advertising, incentive programs, product displays, and exclusive arrangements; 84% of participating schools reported at least one form of marketing, with different patterns in primary and secondary schools (4). These findings locate commercial access within revenue generation, rewards, food provision, and routine school operations.

Product-level and observational studies reveal a gap between nutrition standards and brand promotion. In Vancouver, promotions appeared in every surveyed secondary school and 80% of elementary schools; about one quarter depicted products prohibited for sale under provincial guidelines, and nearly one third promoted commercial items (7). A US assessment of a large school brand-reward program found that fewer than one third of participating entrées and snacks met federal Smart Snacks standards (8). Standards for the nutritional composition of products sold do not necessarily cover corporate logos, sponsorship and supply relationships, or fundraising through branded-product sales and incentives (4). Here, brands comprise product and corporate names, logos, and related identifiers; corporate relationships comprise sponsorship, supply agreements, and branded reward programs. Fundraising arrangements generate institutional income or goods through branded-product sales or purchase incentives.

### 3.2 Organizational rules and implementation

Implementation means translating formal restrictions into procurement and sponsorship conditions, assigning responsibility, communicating requirements, monitoring compliance, and correcting violations. State policy guidance was positively associated with some district advertising restrictions, whereas technical assistance was negatively associated with two restrictive practices and positively associated with one healthy-promotion practice (9). Superintendents identified fundraising, budget constraints, and parent-group relationships as persistent barriers (10). In the Philippines, limited resources, ambiguous definitions, and weak monitoring impeded implementation (11). Stakeholders in Greater Accra, Ghana, assessed community readiness to address unhealthy food marketing and availability as preplanning (12). In Maine, an audit conducted after a statewide marketing ban found noncompliant advertising in 85% of 20 high schools; administrators most often requested implementation resources and clearer guidance (13). In Uruguay, school-principal accounts and observations identified company visits, promotional activities, and branded refrigerators within a healthy-snacking policy environment (14). Repeated surveys of Minnesota secondary schools found that prohibitions on unhealthy-food advertising on school grounds became less common between 2008 and 2012 (15).

### 3.3 Purchasing, dietary, and weight outcomes

Dietary findings vary across designs and settings. A review of 27 school-marketing papers identified five studies of diet-related outcomes; three reported associations with purchasing or consumption, particularly for minimally nutritious products (2). In Minnesota, linked school-policy and adolescent survey data provided little consistent evidence of dietary differences associated with advertising bans or healthier-food promotion (16). In Brazil’s ERICA study, school soft-drink advertising was not significantly associated with sugary-drink intake in either public or private schools, while on-site sales showed positive associations (17). In 12 Addis Ababa schools, school advertising was not significantly associated with dietary diversity, healthy or unhealthy dietary scores, or BMI-for-age among 217 adolescents aged 15–19 years (18). The scoping review by Farmer et al. (19) also covers nutrition-promotion activities, making the commercial exposure assessed in each primary study important to interpretation.

Chile provides evidence on combined regulation: warning labels, marketing restrictions, and school-sales rules were followed by changes in household purchases and school-time intake (20, 21). Weight evaluations address different comparisons and follow-up periods. Repeated grade-level surveillance from 2013 to 2019 showed no sustained decline in overweight or obesity after the 2016 law (22). Using linked records for 321,597 children aged 4–6 years from the same surveillance system, a cohort difference-in-differences study estimated lower excess-weight probabilities after 18 months of exposure (23). Interpretation depends on cohort comparability: pre-trends could not be tested for the 18-month cohort, and the girls’ BMI-for-age comparison showed marginal evidence against parallel pre-trends. These evaluations concern the integrated policy package, with institutional rules operating alongside its other components.

## 4 Youth sport: sponsorship, contracts, and brand responses

### 4.1 Commercial access and exposure

Youth-sport sponsorship links commercial brands with physical activity, teamwork, and community participation. An early systematic review identified limited and geographically concentrated research on food environments and marketing in sports settings (1). Australian club research documented company signage on uniforms, branded rewards, and product vouchers; for most clubs, total sponsorship contributed less than one quarter of income (24). Observational studies subsequently documented food-company sponsorship and geographic differences across children’s clubs (25–27).

Recent studies describe how agreements and institutional assets relate to exposure. Among 85 Canadian facilities, marketing was more frequent where sponsorship agreements existed; the study also reported an association with food-service contracts (5). A related manager survey documented cash and in-kind contributions exchanged for signage, branded materials, equipment, and exclusive rights (6). Photo analysis across 134 facilities identified 2,576 marketing instances. Among the 1,846 instances assessed for healthfulness, 1,583 (85.8%) were classified as nutritionally ‘of concern’; branded infrastructure, displays, and furniture were common promotional vehicles (28). These three reports draw on the same facility project, with different analytic samples.

Contractual access also reaches youth programs hosted by universities. Among 139 beverage contracts from 132 U.S. public universities, 15 addressed youth athletic camps and three addressed high-school athletic events, with provisions exchanging products, branded merchandise, or support for promotional opportunities (29). These child-serving provisions identify promotional rights within a wider university contract sample.

Children’s reported advertising exposure also varied across participation and social contexts in Canada; exposure was reported in Quebec as well as in other provinces (30).

### 4.2 Organizational rules and implementation

The validated FoodMATS approach measures marketing frequency, product healthfulness, child targeting, sport-related appeals, and physical scale (31). In a facility audit, voluntary nutrition guidelines were associated with a lower proportion of least-healthy marketing but not lower marketing frequency (32). A capacity-building intervention did not show an overall improvement in marketing practices attributable to capacity building (33). In the more recent contract study, the presence of a sponsorship policy was not associated with lower marketing frequency (5). These findings distinguish formal policy adoption from changes in institutional promotion.

### 4.3 Brand responses and sponsorship acceptability

Brand-response studies measured sponsor recall, attitudes, and product preferences. Junior players recalled sponsors, described them positively, and reported receiving branded performance rewards (34). In an Australian experiment, healthier-food or public-health sponsorship reduced preference for some unhealthy sponsor products, while unhealthy sponsorship produced no significant change in the measured child brand responses (35). A separate parent experiment found increased awareness, favorable attitudes, and product preference following unhealthy sponsorship (36). Both experiments used simulated sponsorship exposures and measured brand-related responses rather than dietary intake.

Qualitative findings show a different aspect of sponsorship: children in Ontario soccer and hockey said that they would continue playing without food rewards, and participants did not identify a conscious sponsorship influence on treat choices or motivation (37). These accounts describe perceived motivation and coexist with appreciation of sponsors’ financial support.

Parents rated healthier-food brands and health-promotion campaigns as more appropriate sponsors of junior sport than unhealthy-food brands on a seven-point sponsorship-appropriateness scale (36). Surveys and interviews also identified concern about unhealthy sponsorship alongside funding and implementation barriers among parents and club officials (38). Sponsor acceptability and financial feasibility therefore represent distinct aspects of organizational change.

### 4.4 Purchasing-related outcomes

Within the Eat Play Live facility sample, marketing scores were associated with least-healthy concession sales and combined food-service sales after adjustment for facility size; the model for vending sales was not significant. Analyses used 30 concession sites, 23 vending sites, and 21 sites with both sources of sales data (31). These facility-level sales measures concern purchasing, not individual children’s intake. The validation, baseline audit, and intervention reports draw on the same facility framework.

## 5 Cross-setting controversies and policy options

The following policy options derive from our interpretation of the reviewed findings through the institutional-gatekeeping framework.

First, institutional benefits create a tension with commercial access. School leaders and club officials identified budget constraints and transition barriers (10, 38). Total sponsorship provided less than one quarter of income for most surveyed Australian clubs (24), whereas New Zealand regional rugby managers emphasized its importance to service delivery (39). In Ireland, 38% of 331 school respondents accepted for-profit sponsorship, predominantly citing insufficient equipment funding; these measures covered sponsors across industries (40). Financial reliance therefore varies, making contract value and replacement cost relevant to policy design.

One proposed implementation pathway connects sponsor and asset rules with local contract clauses, agreement registers, and compliance audits. Its rationale draws on documented implementation and contractual constraints (5, 6, 9, 11, 33, 38). Exposure frequency, legacy-asset removal, replacement income, and participation costs offer measurable evaluation criteria.

Second, restriction and substitution have different implications. Healthier-food or public-health sponsorship can improve acceptability and selected brand responses (35, 36). Transition options include sponsor eligibility standards, non-food or public-health replacements, pooled sponsorship, grants, and contract phase-outs. Portfolio-level assessment could address continued corporate-brand promotion through a qualifying product.

Third, institutional assets and brand promotion warrant attention alongside product rules. Only 16% of countries in a review of policies collected in 2022–2023 had national school food-marketing restrictions; monitoring and enforcement guidance was often absent (41). Digital recommendations identify content filtering, instructional materials, student-owned devices, and institutional communications as intervention points (42). Extending rules across fundraising, uniforms, equipment, and service contracts is a further policy option.

Brand-level rules require a defined regulatory unit: product, brand, or corporate portfolio. Wider coverage could address brand-only promotion but increases administrative demands.

Fourth, equity concerns both exposure and implementation. Reviews and audits report socioeconomic or geographic differences in school marketing and sport sponsorship, alongside disparities in sport participation (2, 26, 30). Restrictions without replacement resources could increase fees or reduce programs in lower-resource institutions. Evaluations therefore need to track exposure, replacement income, participation costs, and enforcement capacity together.

Table 1 separates institutional arrangements and measured outcomes from proposed policy levers across the two settings.

**Table 1:** Institutional pathways, measured outcome domains, and proposed policy options in schools and youth sport.

Setting	Institutional gatekeepers	Commercial-access channels	Resource dependency or constraint	Outcomes assessed in reviewed publications	Proposed policy levers
Schools	Boards, districts, administrators, parent organizations, food-service and procurement staff	Fundraising, incentives, branded programs, signage, vending, product displays, digital platforms, exclusive agreements	Program revenue, parent-group relationships, administrative capacity, ambiguous rules	Marketing presence and product nutritional profile; purchasing, intake and BMI assessed in a smaller subset; contextual policy-package evaluations also assess child weight	Comprehensive marketing definitions, brand-level standards, contract disclosure, district monitoring, compliant fundraising alternatives
Youth sport	Governing bodies, clubs, coaches, municipal facility managers, food-service contractors	Sponsorship, uniforms, equipment, awards, vouchers, facility signage/furniture, concessions, service contracts	Cash and in-kind support, participation costs, rural sponsor scarcity, legacy assets	Marketing presence and product nutritional profile; sponsor recall, attitudes, preferences, acceptability, and facility-level sales	Sponsor eligibility, replacement funding, pooled healthy sponsorship, contract clauses, facility audits, protection across age and asset types

## 6 Discussion

The first review question concerns commercial access. Across schools and youth sport, the literature documents fundraising, incentive programs, sponsorship, organizational assets, and contracts as access channels. Rules confined to product sales can leave sponsorship rights or legacy promotional assets outside their scope.

For the second question, reported funding concerns and exchanges of cash or in-kind support for promotional rights suggest how resources could sustain access (6, 10, 38). Where alternatives are costly or unavailable, accepting or renewing an agreement can preserve institutional activities while retaining brand exposure. This is an interpretation of the documented arrangements, not a directly demonstrated causal effect of resource dependence. Associations between agreements and marketing frequency support examining this pathway, while null policy and intervention findings show that formal adoption alone is insufficient evidence of change (5, 33).

Two testable expectations follow: dependence on commercial support may accompany greater exposure where rules omit promotional rights, and broader restrictions combined with replacement resources and monitoring may reduce exposure over time. Agreement values, replacement costs, and repeated audits would allow these propositions to be tested.

For the third question, evidence is comparatively stronger for marketing presence, nutritional characteristics, brand recognition, attitudes, and selected preferences. Purchasing and intake findings are more limited and heterogeneous, and longer-term health evidence concerns broader policy packages rather than isolated institutional marketing. Institution-specific longitudinal measures are needed to connect these domains.

Evaluations could combine channel inventories, contracts, nutrient profiles, repeated purchasing or intake measures, and implementation costs. Natural experiments distinguishing marketing from labeling, sales, and procurement reforms would help identify the contribution of institutional restrictions.

Proportionality means matching restrictions to expected exposure reduction while considering administrative costs and children’s participation. Substitution raises a separate question: whether healthier sponsor products continue to promote a wider corporate brand. Both trade-offs require assessment alongside replacement income and participation fees.

All 42 included publications were verified against the original sources, but only some records not selected by GPT-5 were manually checked. Relevant publications may therefore have been missed during AI-assisted selection. Restricting the evidence map to Web of Science, Scopus, and English-language journal publications can omit government and regulatory documents, contracts, organizational reports, and non-English evidence. The absence of formal risk-of-bias or certainty assessment limits comparisons across cross-sectional, qualitative, experimental, audit, policy-evaluation, and review evidence. Much detailed institutional evidence comes from Canada and Australia; resource availability, contractual arrangements, school governance, and sponsorship structures differ across policy systems, limiting transfer of the proposed framework without contextual assessment.

## 7 Conclusion

Schools and youth-sport organizations govern commercial access through procurement, fundraising, sponsorship, assets, and contracts. The synthesis identifies these channels and selected brand responses more clearly than their dietary or long-term health consequences. Resource dependence offers an explanation to test, while broader access rules, contract transparency, monitoring, and replacement resources constitute policy options for evaluation.
