# Supplementary material for: Institutional gatekeeping of food and beverage marketing in schools and youth sport
Source: Front Nutr. 2026 Oct 19;13:1953630. doi: 10.3389/fnut.2026.1953630 (PMC13760620; doi:10.3389/fnut.2026.1953630)
Supplement: Supplementary file 1 [file Table_1.DOCX]

Supplementary search strategies and record accounting

Supplementary Table S1. Executed database queries and common record-count stages.

# Web of Science Core Collection

TS=(((food* OR beverage* OR drink*) NEAR/3 (marketing OR advertis* OR brand* OR sponsor*)) AND (child* OR adolescen* OR youth* OR teen* OR "young people" OR preschool*) AND (school* OR childcare OR "child care" OR daycare OR "day care" OR nursery OR ((youth* OR junior* OR school* OR community*) NEAR/2 sport*) OR (sport* NEAR/2 (club* OR setting* OR facilit*)) OR (recreation* NEAR/2 facilit*))) AND PY=(2000-2026)

# Scopus

TITLE-ABS-KEY(((food* OR beverage* OR drink*) W/3 (marketing OR advertis* OR brand* OR sponsor* OR "sales promotion*" OR "commercial promotion*")) AND (child* OR adolescen* OR youth* OR teen* OR "young people" OR preschool* OR pupil*) AND (school* OR kindergarten* OR childcare OR "child care" OR daycare OR "day care" OR nursery OR ECEC OR "early childhood education and care" OR ((youth* OR junior* OR school* OR communit*) W/2 sport*) OR (sport* W/2 (club* OR setting* OR facilit* OR team* OR event* OR league*)) OR (recreation* W/2 facilit*))) AND PUBYEAR > 1999 AND PUBYEAR < 2027 AND (DOCTYPE(ar) OR DOCTYPE(re)) AND SRCTYPE(j) AND LANGUAGE(english)

Search date: 29 July 2026. The displayed Web of Science history used community* after parsing the entered communit*. The original queries and exports are retained without retroactive alteration.

| **Record-count stage** | **Web of Science** | **Scopus** |
| --- | --- | --- |
| Query hits at recorded platform stage | 757 before Article/Review export filter | 731 after all query-level limits |
| Exported records | 736 | 731 |
| English-language records after offline check | 695 | 731 |
| Records after exact offline year, language and document-type validation | 675 | 731 |
| Combined validated records before deduplication | 1,406 across both databases |  |
| Unique records after DOI/normalized-title deduplication | 893 across both databases |  |

The first row records the actual platform stages; an unfiltered Scopus hit count was not recorded. The combined exports totalled 1,467 records. The archived exact-string document-type filter removed 20 compound-type Web of Science records after the English-language check: 11 proceedings papers, five book chapters, one data paper, one retracted publication, and two early-access records. The two early-access articles were retained through their Scopus records in the 893-record map; early-access status itself is not a non-peer-review criterion. Deduplication removed 513 records from the validated set. These stages describe the archived processing.
