# Supplementary material for: Institutional gatekeeping of food and beverage marketing in schools and youth sport
Source: Front Nutr. 2026 Oct 19;13:1953630. doi: 10.3389/fnut.2026.1953630 (PMC13760620; doi:10.3389/fnut.2026.1953630)
Supplement: Supplementary file 2 [file Table_2.DOCX]

Supplementary evidence map

**Supplementary Table S2. Characteristics, measured findings, and thematic contributions of the 42 publications cited in the revised narrative.**

T1 = commercial access and exposure; T2 = organizational rules, resources, and implementation; T3 = brand responses and sponsorship acceptability; T4 = purchasing, dietary, and nutrition-related health outcomes. Themes describe content, not evidence strength. Role identifies how each publication contributes to the synthesis. Direct denotes empirical studies of institutional marketing arrangements, exposure, implementation, or measured responses and outcomes in school- and youth-sport-related settings. Experiment denotes the two simulated junior-sport sponsorship studies examining immediate child or parent responses. Context denotes reviews, broader policy and organizational analyses, and combined-policy evaluations that inform interpretation of field-level patterns, the institutional pathway, and policy implications. Each publication’s specific contribution is described in the “Commercial arrangement and narrative contribution” column. NR = not reported in the examined source.

| **Publication and role in the synthesis** | **Design, setting, and sample** | **Commercial arrangement and narrative contribution** | **Measured findings** | **Theme codes** |
| --- | --- | --- | --- | --- |
| FG-0001 Prowse et al. (2025) Direct | Cross-sectional comparison of FoodMATS facility audits with facility-manager survey reports. Nine Canadian provinces; publicly funded indoor recreation/sport facilities with skating, swimming or soccer amenities used by children. Schools, seasonal/outdoor facilities and facilities on Indigenous land or north of 55° latitude were excluded. 134 facilities audited; 86 manager surveys; 85 facilities with linked audit/survey data analysed, containing 2,429 marketing instances. | Food-related sponsorship policies, sponsorship agreements and food-service contracts; observed marketing was matched to manager-named sponsors or contract companies.  **Narrative contribution:** Marketing counts by policy, sponsorship and service-contract status; proportion of observed marketing matched to named companies. | Marketing frequency was similar with versus without sponsorship policies (medians 14.5 versus 18.0; p=.37), but higher with sponsorship agreements (26.5 versus 12.5; p<.001). Within relevant facilities, 305/1,364 marketing instances (22.4%) matched named sponsors and 368/2,191 (16.8%) matched named contract companies. Linked audit/manager reports from 85 facilities. Internally inconsistent contract counts and medians preclude a reliable exact contract-effect estimate. | T1 T2 |
| FG-0002 Scully et al. (2020) Experiment | Online randomised between-subject experiment with four sponsorship conditions. Australia; hypothetical Go Team junior multisport programme for children aged 6–9, evaluated by parents. 1,331 parents: non-food sponsor n=330; unhealthy-food sponsor n=334; healthier-food sponsor n=334; public-health campaign n=333. | A programme video and flyer displayed experimentally assigned sponsor branding; a free branded sports pack was also shown.  **Narrative contribution:** Parent sponsor awareness, brand attitudes, programme ratings and product-choice task. | Unhealthy sponsorship increased parent awareness (36.5% versus 21.4%) and unhealthy-product selection (31.2% versus 21.5%). Healthier-food and public-health sponsorship increased healthier-product selection (36.5% and 36.1% versus 28.2%). Healthier-brand attitudes, public-health campaign awareness, programme interest, expected enjoyment, enrolment likelihood and sports-pack appeal did not differ significantly. Randomized simulated junior-sport programme with 1,331 parents; immediate product-choice task rather than observed purchasing. | T3 |
| FG-0008 Dixon et al. (2018), Public Health Nutrition, 21(6), 1176–1185 Experiment | Randomized online experiment with four simulated junior-sport sponsorship conditions and three product categories. Melbourne, Australia; administered through primary schools but stimuli simulated joining a local sports club and receiving sponsor-branded merchandise. 1,124 children aged 5–10 years in grades 1–3 across 12 schools; reported student participation 44% and school participation 10%. | Non-food control, unhealthy-food sponsor, healthier-food sponsor, or obesity-prevention campaign on simulated junior-sport merchandise.  **Narrative contribution:** Sponsor awareness, brand attitudes, and branded-product preferences immediately after exposure; no actual purchasing or intake. | Unhealthy-food sponsorship did not increase unhealthy-brand awareness, attitudes or preference versus control; awareness OR .83 (95% CI .55–1.26), preference OR .74 (.51–1.06). Healthier sponsorship increased healthier-brand awareness (OR 1.72, 1.10–2.69), but not preference (OR 1.22, .82–1.81). Healthier-food and obesity-prevention sponsorship reduced unhealthy-product preference. Randomized, brief simulated junior-sport exposure in 1,124 children; immediate brand responses. | T3 |
| FG-0009 Prowse et al. (2018a), International Journal of Behavioral Nutrition and Physical Activity, 15, 38 Direct | FoodMATS inter-rater reliability study plus cross-sectional construct/predictive-validity analyses relating site scores to sponsorship and food sales. Canadian public recreation/sport facilities in four provinces; Eat Play Live study. Five facilities for reliability testing; 51 sites from 49 facilities for baseline audits. Food sponsorship dollars available from 18 facilities; final sales samples: 30 concessions, 23 vending sites, and 21 sites with both. | Facility-wide food marketing scored for frequency, healthfulness, child/sport appeals, and size; linked with food sponsorship revenue and food-service sales.  **Narrative contribution:** Measurement reliability, sponsorship correlations, and least-healthy concession/vending sales; not individual child dietary intake. | Marketing scores correlated with food-sponsorship dollars (size-adjusted r=.863, p<.001), but not total sponsorship. Scores explained an additional 13.8% of least-healthy concession-sales variation (n=30; p=.012) and 23.5% of combined-sales variation (n=21; p=.003). Vending prediction was nonsignificant (n=23), as was the association between food-area scores alone and concession sales (p=.057). Cross-sectional tool-validation and sales-association study; sales are facility-level, not individual child intake. | T1 T4 |
| FG-0011 Velazquez et al. (2017) Context | Systematic review of cross-sectional school food-marketing studies; five databases and six grey-literature sources searched in July 2015, with supplementary identification of studies published before 2016. Schools in the United States, Canada, Ireland, and Poland; predominantly U.S. evidence. 27 papers representing 20 unique data sources; 23 papers from the United States. No new participant sample. | School advertising, commercial educational/incentive activities, and product-sale arrangements including exclusive beverage contracts.  **Narrative contribution:** Extent and measurement of school marketing; associations with purchasing, intake, meal participation, and weight in a small subset of studies. | Three of five studies assessing purchasing/consumption reported positive associations. Other findings were null for advertising-ban scores versus school-average intake and logo exposure versus overweight/obesity; an inverse association with school-lunch participation was marginal (p<.07). Systematic review of 27 papers/20 unique data sources; cross-sectional evidence and heterogeneous exposure measures. | T1 T2 T4 |
| FG-0016 Prowse et al. (2020) Direct | Exploratory secondary-outcome analysis of an 18-month capacity-building randomised trial embedded in a natural experiment; baseline November 2015–May 2016, follow-up August–December 2017. Canadian recreation facilities: British Columbia, Alberta and Nova Scotia had voluntary provincial nutrition guidelines; Ontario was the non-guideline comparator. 49 facilities represented 51 measurement sites. Guideline provinces: capacity building 17 facilities/18 sites, guideline-only 15 facilities/16 sites; Ontario: 17 facilities/sites. Complete all-area analyses used 46 sites (15/15/16). | Observed commercial food marketing on facility surfaces and equipment; facility action plans included healthy promotion, sponsorship restrictions and marketing policy requirements.  **Narrative contribution:** FoodMATS composite score; frequency, repetition, healthfulness, child-targeting, sport techniques and marketing size. | Capacity building did not significantly improve overall marketing compared with guidelines alone. The only significant between-group change was frequency (p=.045): median +6 instances without guidelines versus 0 in each guideline group; capacity-building versus guideline-only p=.367. FoodMATS and least-healthy-marketing changes were nonsignificant. Complete all-area analyses used 46 sites (15/15/16). Exploratory trial outcome; only within-guideline-province comparisons were randomized. Ontario was a nonrandomized comparator; overall marketing totals may include alcohol-related retailers. | T1 T2 |
| FG-0023 Prowse et al. (2018b), International Journal of Behavioral Nutrition and Physical Activity, 15, 39 Direct | Cross-sectional facility audit using FoodMATS, comparing provinces with and without voluntary nutrition guidelines. Canadian public recreation/sport facilities: Alberta, British Columbia, Nova Scotia, and Ontario. 51 sites from 49 Eat Play Live facilities; 34 sites in guideline provinces and 17 in Ontario without guidelines; 1,740 marketing occasions. Two facilities each contributed two separate buildings. | On-site product/brand/retailer marketing across food-service, sports, and general facility spaces, including child-targeted and sport-related techniques.  **Narrative contribution:** Marketing frequency/repetition, healthfulness, size, location, and persuasive techniques; not child consumption. | Marketing appeared at 50/51 sites; 55.6% of 1,740 occasions promoted least-healthy items. Frequency and repetition did not differ by guideline status (p=.576 and .217). Guideline provinces had less least-healthy marketing (47.9% versus 73.5%) but more child-targeted and sports-related techniques. Cross-sectional provincial comparison of voluntary guideline settings. | T1 T2 |
| FG-0030 Potvin Kent et al. (2019) Direct | Cross-sectional online principal survey, spring 2016. Canada; public schools in British Columbia, Ontario and Nova Scotia. Provincial comparisons excluded the small Nova Scotia group. 154 schools: 108 primary and 46 secondary, from 27 participating school boards. Board participation was 27/145 (18.6%); this is not the principal response rate. | Branded fundraising, advertising on school property, incentives, exclusive marketing arrangements and commercial materials.  **Narrative contribution:** Marketing prevalence/types, policy awareness and attitudes to commercial exchange; no student diet. | Of 154 schools, 84% reported food marketing, including branded fundraising (64%), on-property advertising (26%) and incentives (18%). Eighteen percent considered marketing an acceptable trade-off for resources and 54% disagreed; 83% rejected the view that commercial motives were irrelevant when schools benefited. Principal self-reports in three provinces; 27/145 school boards participated. | T1 T2 |
| FG-0031 Pauzé et al. (2020) Direct | Cross-sectional audit of sports-club websites and linked newsletters, September–December 2018. Ottawa, Ontario, Canada; clubs offering soccer, swimming, ice hockey, basketball or baseball to members younger than 18. 67 eligible clubs; 312 sponsorship instances, including 51 food/beverage sponsorship instances representing 31 different companies. Instances are not unique sponsors. | Club website acknowledgement, logos and sponsor-related promotion; sponsorship operationalisation also included branded events, fundraisers, discounts, coupons and prizes.  **Narrative contribution:** Prevalence and type of food-company sponsorship by club and sponsor category. | Food/beverage sponsors appeared at 27/67 clubs (40%), including fast-food sponsors at 17 (25%). Food/beverage sponsorship accounted for 51/312 instances (16.3%), of which 23/51 (45%) were fast-food companies. Website/newsletter audit; sponsorship instances are not unique sponsors. The food/beverage aggregate includes one alcohol instance. | T1 |
| FG-0049 Bragg et al. (2018) Context | Narrative review of sports-related food and non-alcoholic beverage marketing. International examples across professional, local and junior sport, athlete endorsements and sports-themed media/games. NR: no total included-study count or new participant sample reported in the examined narrative-review material. | Food-brand sponsorship of teams and junior clubs, athlete endorsements, branded events and sports-linked promotions.  **Narrative contribution:** Prior evidence on exposure, sponsor recall, brand attitudes and preferences; policy arguments and research gaps. | The review describes food-brand relationships in junior, local and professional sport, including sponsor recall, brand attitudes/preferences and sport-related health cues. Replacement funding and compensation are discussed as policy options. Narrative review; junior-sport evidence sits alongside elite-sport and media studies. | T1 T3 |
| FG-0055 Prowse et al. (2026) Direct | Cross-sectional manager survey of food-related sponsorship and service contracts. Nine Canadian provinces; publicly funded indoor recreation/sport facilities, almost all hosting child teams. 86 facilities with survey data through the sponsorship section; 83 fully completed surveys. Item-specific denominators exclude missing, refused and unknown responses; counts and percentages therefore do not all use 86. | Food-company cash, equipment, food, vouchers and other contributions exchanged for signage, logos, digital promotion, naming opportunities and exclusive service/product rights.  **Narrative contribution:** Prevalence and value of food sponsorship/contracts; financial/in-kind contribution types and marketing benefits. | Twenty-seven facilities reported food sponsorship and 42 service contracts. Among 44 sponsors with financial details, 39 (88.6%) provided cash; median contribution was CAD 500 (IQR 288–1,375). Financial data covered 16 facilities; food sponsors supplied a median 25% (IQR 13.9–83.3%) of sponsorship income among recipients. Benefits included signage, branded materials, equipment and exclusivity. Retrospective manager reports; item-specific prevalence denominators are internally inconsistent. The 25% share concerns sponsorship income, not total facility income. | T1 T2 |
| FG-0056 Wilking et al. (2023) Context | Policy/practice analysis: literature and law review plus stakeholder telephone input United States; school networks, school-issued devices and digital materials Stakeholder interview count NR in examined text; no new student outcome sample | School-approved digital content, device filtering, vendor contracts and school communications  **Narrative contribution:** Supplies a full-text policy analysis of school-controlled digital materials, devices and communications; used for policy options rather than as an empirical exposure evaluation. | The analysis proposes food-marketing safeguards in four school-policy areas: content filtering, instructional materials, student-owned device use and communications. Vendor contracts and school-controlled digital infrastructure provide potential implementation routes. Policy/practice analysis with model language; recommendations were not evaluated as an intervention. | T2 |
| FG-0058 Lei et al. (2026) Direct | Cross-sectional secondary content analysis of photographs from facility FoodMATS audits, February–May 2022. Nine Canadian provinces; publicly funded indoor recreation/sport facilities providing skating, swimming or soccer, used by children and adults. 134 facilities; 3,521 observed marketing instances, of which 942 had missing/unclear photographs and three were non-commercial, leaving 2,576 coded advertisements. Healthfulness analysis used 1,846 after excluding 346 unclassifiable brand/retailer ads and 384 grocery/agriculture/alcohol ads. | Food/beverage advertisements on facility surfaces and equipment; coding of general persuasive and sport-related techniques, including brand infrastructure.  **Narrative contribution:** General/sport-related technique prevalence; association between sport techniques and advertised-item healthfulness. | The Results reports general techniques in 2,354/2,576 advertisements and sport techniques in 309/2,576 (12.0%). Nutritional concern applied to 1,583/1,846 classifiable advertisements (85.8%). Sport-related advertisements more often concerned poor nutrition (97.6% versus 84.6%; p<.001); general-technique presence was unrelated to healthfulness (85.4% versus 89.6%; p=.171). Photo content analysis. Results denominators are retained; abstract percentages and a non-sport count are inconsistent. Alcohol was excluded only from healthfulness analysis. | T1 |
| FG-0087 Polacsek et al. (2012) Direct | Cross-sectional observation and administrator interviews, spring 2010. Maine, United States; public high schools. 20 schools selected through urban/rural stratified random sampling, with replacement of three nonparticipating schools. | School signs, scoreboards, vending exteriors, sponsorships, incentives and fundraising under a statewide marketing restriction.  **Narrative contribution:** Observed noncompliance, policy knowledge, implementation needs and reported revenue changes; no dietary outcomes. | Eighty-five percent of 20 high schools were noncompliant, averaging 12 marketing instances per school. Administrators requested resources in 80% of schools and funding specifically in 30%; 15% reported lower revenue. Cross-sectional audit with administrator interviews; revenue changes were self-reported. | T1 T2 |
| FG-0089 Pauzé et al. (2025; Health Promotion International) Direct | Cross-sectional analysis of the 2019 International Food Policy Study Youth Survey, using weighted regression. Canada; online survey in November–December 2019 of children aged 10–17, recruited through parents in a commercial panel. 3,825 completed responses; 143 removed for eligibility/quality and 10 for missing sport data, yielding 3,672 analysed children. The weighted team-sport subgroup was 1,451 (39.5%); this is not an independent raw recruitment count. | Self-reported food-brand names/logos on sports equipment such as uniforms/water bottles and on signs/banners where children practised, played or competed.  **Narrative contribution:** Team-sport participation; reported brand exposure across two channels; sociodemographic and provincial associations. | Among team-sport participants, 34.1% reported food-brand signs, 22.0% branded equipment and 40.2% either channel. Compared with Quebec, exposure odds outside Quebec were higher for signs (OR 1.39, 95% CI 1.04–1.84) and equipment (1.49, 1.06–2.09). Equipment exposure was higher among children reporting inadequate income. Weighted cross-sectional self-reports; 3,672 analyzed children, weighted sport subgroup 1,451. Unknown exposure was coded unexposed. | T1 |
| FG-0093 Asada et al. (2020), Public Health Nutrition, 23(11), 2024–2031 Direct | Qualitative focus groups and follow-up telephone interviews. United States; public K–12 school districts represented at the March 2017 AASA annual meeting. 39 superintendents/assistant superintendents from 23 states and all four Census regions; 14 participated in follow-up interviews. Convenience recruitment through a professional meeting. | Restaurant fundraisers, branded school communications, banners, and restaurant-linked academic rewards; on- and off-campus activities connected to schools/PTA/PTO groups.  **Narrative contribution:** Administrator awareness, perceptions, reported policies, fundraising practices, and oversight; no measured child dietary effects. | Most administrators were unaware of the forthcoming marketing-policy requirement three months before its deadline. Practices ranged from restrictive policies to limited oversight. Restaurant fundraisers were common, and parent-group autonomy, budget pressures and after-hours exceptions complicated implementation; some administrators questioned the financial return. Qualitative reports from 39 superintendents/assistants; findings describe practices and perceptions, not national prevalence. | T1 T2 |
| FG-0105 Carter et al. (2012) Context | Systematic review of English-language studies published in 1985–2011; final journal issue 2012, first online 2011. Sports clubs, stadia, websites and televised sport; Australia (10 reports), United Kingdom, New Zealand, United States and Canada (one each). 14 reports; two describe different aspects of the same study. Includes observational, interview, focus-group and survey evidence; no new sample. | Food/beverage sponsorship, branded equipment and uniforms, vouchers, discounts, club-linked promotion and food availability.  **Narrative contribution:** Availability and sponsorship patterns, stakeholder views, and club policy practices; evidence on children's dietary effects was limited. | Club sponsorship commonly provided minor or in-kind support. For most clubs, all sponsorship contributed less than one-quarter of income. Reviewed club-policy interventions largely involved health-promotion funding; evidence on dietary effects was limited. Review spans clubs, stadia and televised sport. Income estimates concern all sponsors, not unhealthy-food sponsors alone. | T1 T2 T3 |
| FG-0106 Farmer et al. (2025) Context | Scoping review; search in January–February 2024 for studies from 2011–2023; eligibility required food-choice/consumption relevance. School-environment literature from India, Australia, Wales, Austria, Guatemala, Portugal, Indonesia, Zambia, the United States and Canada. 12 studies reported from 1,608 references; Table 1 contains a duplicate row for Jonatan et al. (2022). | Mixture of commercial advertising/branding and wider school nutrition interventions, including fruit/vegetable nudges, salad-bar promotion and lunch samples.  **Narrative contribution:** Heterogeneous food choices, consumption, perceptions and brand responses. | The 12 included studies combine commercial food marketing with noncommercial nutrition initiatives. Dietary findings across this broad intervention set were positive or null, whereas the commercial-sponsorship experiment primarily measured brand responses. Scoping review with mixed intervention types; Dixon et al. concerns simulated junior-sport sponsorship, not school marketing. | T1 T4 |
| FG-0113 Perry et al. (2024) Context | Global policy mapping of national mandatory competitive-food and school food-marketing policies; collection August 2022–June 2023. 193 countries; preschool through secondary school. Subnational and voluntary policies were outside the national-policy inventory. 193 countries assessed; 30 had national school-marketing policies, 48 competitive-food policies, and 54 either type. | National restrictions on school food/beverage marketing, including coverage of marketing forms and locations.  **Narrative contribution:** Policy existence, coverage, comprehensiveness and implementation provisions; not observed advertising exposure or child diet. | Thirty of 193 countries (16%) had national mandatory school-marketing policies: nine total and 21 partial bans. Of these 30 countries, 20 specified monitoring, 14 enforcement, and 12 both. Policy collection: August 2022–June 2023. Counts describe written national provisions, not observed enforcement. | T2 |
| FG-0122 Kelly et al. (2011a), Health Promotion International, 26(2), 188–195 Direct | Cross-sectional telephone survey of randomly sampled sports clubs, with expert-derived healthy-sponsor criteria. Australia; nine popular children's sports across Sydney, Illawarra, and Canberra/Queanbeyan areas. 108 of 109 clubs responded (99%); 347 sponsors, including 58 food/beverage sponsors. The income analysis concerns 70 clubs receiving any sponsorship. | Club and association sponsorship exchanged funding/in-kind support for uniform logos, signage, product sale/use, and branded sporting rewards.  **Narrative contribution:** Sponsor type/healthfulness, promotional benefits, support provided, proportion of club income from all sponsorship, and written sponsorship policies; no child dietary outcomes. | Food/beverage businesses comprised 58/347 sponsors (17%); half of their arrangements failed healthy-sponsor criteria. Among 58 sponsors, 53% received uniform signage, 24% branded rewards and 28% product sale/use; 41% supplied funding and 29% vouchers. Among 70 sponsored clubs, 67% obtained less than one-quarter of income from all sponsorship, and 30% had a written policy. Club survey; income and written-policy denominators concern any sponsorship, not unhealthy-food sponsorship alone. | T1 T2 |
| FG-0137 Zorbas et al. (2023) Direct | Mixed-methods study: cross-sectional parent panel survey and semi-structured interviews with purposively sampled club officials, 2020. Victoria, Australia; junior community sports clubs in urban and regional areas. 504 eligible parent survey respondents; 16 officials from 16 clubs already accepting unhealthy-food sponsorship. Officials comprised 12 presidents, two secretaries, one sponsorship coordinator and one treasurer. | Negotiated or packaged sponsorship deals exchanging funds, equipment and player food vouchers for ground signage, uniform logos, social-media promotion and other club communication.  **Narrative contribution:** Parent-reported sponsorship exposure/reward use and policy attitudes; official accounts of procurement, funding and transition barriers. | Officials estimated food-sponsor contributions of A$500–10,000 annually and all-sponsor revenue shares of 5–60%. Among 504 parents, 58% were concerned about local and 63% about large fast-food sponsors; 43% would pay higher fees to avoid unhealthy advertising. Reward-use results reported 65% spending beyond rewards. Officials described funding and replacement barriers. Estimated finances in 16 already-sponsored clubs. The denominator for the 65% reward-spending item is unclear in the examined main text. | T2 T3 |
| FG-0149 Kelly et al. (2010) Direct | Census-invitation cross-sectional school survey Republic of Ireland; post-primary schools across all 26 counties 331 of 741 invited schools responded (44.7%); principal/staff reports, no student sample | For-profit sponsorship, equipment funding, school food outlets and commercial policies  **Narrative contribution:** Adds school sponsorship and resource context from Ireland, with the cross-industry denominator identified. | Thirty-eight percent of responding schools accepted for-profit sponsorship, and 7% had a commercial-sponsorship policy. Inadequate equipment funding was a prominent sponsorship rationale (reported 91.6%). 331 of 741 schools responded (44.7%). Sponsorship measures cover commercial organizations across industries. | T1 T2 |
| FG-0159 Gonzalez et al. (2020) Direct | Cross-sectional analysis of baseline telephone surveys from a broader cluster-randomised Good Sports trial, March–August 2016. New South Wales and Victoria, Australia; junior Australian football and rugby-league clubs with at least 40 players, participating at Good Sports Level 3. 79 club representatives from 79 clubs and 297 parents; eligible/recruited frames were 86 clubs and 387 parent contacts. | Fast-food and alcohol sponsorship; food-specific arrangements included discounted food/drink, cash contributions and vouchers.  **Narrative contribution:** Fast-food/alcohol sponsorship prevalence; sponsorship forms; associations with club characteristics; attitudes to restrictions. | Fast-food sponsorship was reported by 21/79 clubs (27%); these sponsors provided discounts (57%), cash (52%) and vouchers (38%). Restricting fast-food rewards/fundraising was supported by 29/79 representatives (36.7%) and 125/297 parents (42.1%). The adjusted regional association was imprecise (OR 9.1, 95% CI 1–84; p=.05); other adjusted club-characteristic associations were nonsignificant. Baseline survey; some representatives could not distinguish junior-team from whole-club sponsorship. | T1 T3 |
| FG-0164 Rocha et al. (2021) Direct | Cross-sectional multilevel analysis of ERICA school observations and 24-hour recall Brazil; public/private schools in 124 municipalities 1,247 schools; Methods final analytic N=71,475 aged 12–17, while Results/Table 3 print N=71,549; discrepancy retained | Observed school soft-drink advertising and sales  **Narrative contribution:** Adds school-advertising-specific null associations with sugary-drink consumption in Brazil. | School soft-drink advertising was unrelated to intake after adjustment: 5.2 mL/day (95% CI −35.4 to 46) in public schools and −10 mL/day (−44.3 to 21) in private schools. Positive sales associations concerned a separate exposure. Cross-sectional ERICA analysis of 1,247 schools; Methods N=71,475 versus Results/Table 3 N=71,549 remains inconsistent. | T1 T4 |
| FG-0173 Kelly et al. (2011b), International Journal of Behavioral Nutrition and Physical Activity, 8, 95 Direct | Cross-sectional, interview-administered questionnaire with junior sports players; sponsor recall checked against club sources. Australia; food-sponsored clubs across Sydney, Illawarra, and Canberra/Queanbeyan. 103 children aged 10–14 years (mean 12, SD 1.3) from 20 clubs; purposive selection of food-sponsored clubs and convenience recruitment of children; club visits May–November 2010. | Real club sponsorship, branded performance vouchers/certificates, and separately assessed elite-team sponsorship.  **Narrative contribution:** Sponsor recall, attitudes, previous receipt/liking of rewards, and reported purchase-related beliefs/intentions; no measured consumption. | Among 103 children, 68% recalled club sponsors, 86% reported food/beverage vouchers and 76% branded certificates. Among recipients, 30% and 38%, respectively, reported increased company liking. Fifty-nine percent endorsed reciprocating sponsorship by buying products. Younger children expressed stronger reciprocity responses, but age differences in liking after rewards were nonsignificant. Cross-sectional child reports in food-sponsored clubs; purchase-related beliefs and intentions, not measured purchases. | T1 T3 |
| FG-0181 Marx et al. (2022) Direct | Cross-sectional content analysis of public-university pouring-rights contracts United States; youth athletic camps and high-school events hosted at public universities 139 contracts from 132 universities, active in 2018–2019; obtained June 2019–August 2020. No participant sample. | Beverage marketing rights, free drinks and branded merchandise in university-operated youth programs  **Narrative contribution:** Adds directly specified youth-camp and school-related promotional rights in university beverage contracts. | Twenty-two of 139 contracts contained 25 youth-targeted provisions, 24 linked to athletics. Fifteen contracts covered youth athletic camps and three high-school athletic events; categories could overlap. Five youth-camp provisions addressed possible future legal or self-regulatory restrictions. Contract-content analysis of 132 public universities; written youth-programme rights were examined, not their execution. | T1 T2 |
| FG-0211 Martino et al. (2021) Direct | Cross-sectional audit of club websites and Facebook pages, September–November 2019. Victoria, Australia; eight junior sports across 30 communities sampled by urbanity and area disadvantage; clubs served children aged 5–14. 216 sport-club entries representing 191 distinct clubs; 22 multi-sport clubs were counted separately by sport. 2,032 recorded sponsorship arrangements. | Club sponsor listings and branding in apparel, ground signage, newsletters, annual reports and Facebook photographs.  **Narrative contribution:** Prevalence of unhealthy-food, high-risk-food, alcohol and gambling sponsorship by club/sport and geographic characteristics. | Unhealthy-food sponsors accounted for 96/2,032 arrangements (4.7%) and appeared in 25.9% of 216 sport-club entries; high-risk food sponsors appeared in 18.1%. Regional entries more often had unhealthy-food sponsorship than metropolitan entries (32.7% versus 19.6%; adjusted OR 2.1, 95% CI 1.08–4.20). Online audit of 216 sport-club entries representing 191 distinct clubs; these estimates are food-specific. | T1 |
| FG-0242 Larson et al. (2014) Direct | Repeated cross-sectional school policy surveys (2008, 2010, 2012), plus cross-sectional linkage of 2010 school policies to school-average student diet. Minnesota, United States; secondary-school policy and student-survey data. School Health Profiles: 192 schools in 2008, 261 in 2010 and 261 in 2012. Dietary linkage: 206 schools, using sixth-, ninth- and twelfth-grade student data; student N is NR in the examined material. | Principal-reported bans on candy, fast-food and soft-drink advertising in school buildings, grounds, buses and publications; a zero-to-four-location restriction score.  **Narrative contribution:** Policy prevalence/trends and school-average fruit/vegetable and sugar-sweetened-beverage intake. | Advertising bans did not improve overall from 2008 to 2012 (p=.07). In the 206-school dietary linkage, ban scores were unrelated to sugar-sweetened-beverage intake (β=.03, 95% CI −.01 to .07; p=.20) or fruit/vegetable intake (β=−.01, −.03 to .02; p=.60). Repeated policy surveys with a cross-sectional, school-level dietary linkage in 2010. | T2 T4 |
| FG-0256 Trübswasser et al. (2022) Direct | Cross-sectional school audit and adolescent interviews Addis Ababa, Ethiopia; 12 private/government high schools 217 adolescents aged 15–19 (mean 17.2); school compound and 0.5-km neighborhood audits | SSB advertisements within three government-school compounds  **Narrative contribution:** Adds Ethiopian school evidence with null dietary and BMI associations, broadening setting coverage. | Three government-school compounds displayed sugary-drink advertising. School advertising was unrelated to dietary diversity (p=.67), GDR-Healthy (.59), GDR-Limit (.76) or BMI-for-age (.45). Cross-sectional audit/interviews: 12 schools, 217 adolescents. An inconsistent extracted GDR-Healthy coefficient is omitted; reported p values are retained. | T1 T4 |
| FG-0298 Tandoh et al. (2023), BMJ Open, 13, e075166 Direct | Mixed-method community-readiness appraisal: key-informant interviews, qualitative analysis, and anchored readiness scores on a nine-stage scale. Greater Accra, Ghana; marketing and availability of unhealthy foods in and around public basic schools. 18 key informants across six sectors; 11 women and seven men. This is a stakeholder sample, not a count of audited schools or participating children. | Commercial food/beverage marketing and sale in school environments; readiness to govern these activities rather than an evaluation of one sponsorship contract.  **Narrative contribution:** Community readiness and perceived barriers/facilitators; no measured change in marketing exposure or child diet. | Overall stakeholder readiness was 4.44 (SD .98), indicating preplanning. Leadership scored 5.36 (1.60), knowledge of the issue 5.28 (.99), resources 3.64 (.87), and knowledge of existing efforts 3.19 (2.45). Funding, expertise and sellers’ livelihood concerns shaped perceived feasibility. Community-readiness appraisal of 18 stakeholders; scores measure readiness rather than implementation success. | T2 |
| FG-0299 Reeve et al. (2018), Globalization and Health, 14, 8 Direct | Qualitative policy analysis combining semi-structured interviews, policy documents, and company-webpage content analysis; Health Policy Analysis Triangle framework. Philippines; Greater Manila interviews and national Department of Education school-food/marketing policy. 21 key-informant interviews in May 2016: nine national policymakers, three municipal officers, four principals, three food providers, one nutrition researcher, and one food-regulatory representative; supplemented by documents/webpages. | Commercial support for school buildings, libraries, equipment, scholarships, nutrition activities, and sports events accompanied by brand visibility.  **Narrative contribution:** Policy content, implementation barriers, stakeholder interpretations, and reported/documented school branding; routine policy-effectiveness and child-consumption measures were absent. | Interviewees described branded buildings, gym floors and education/sport activities; company webpages corroborated several forms of support. Some principals cited commercial restrictions while describing branded partnerships. Ambiguous rules, limited resources and weak monitoring were identified as implementation barriers. Qualitative policy analysis using 21 interviews, documents and company webpages. | T1 T2 |
| FG-0308 Fretes et al. (2023), International Journal of Behavioral Nutrition and Physical Activity, 20, 40 Context | Longitudinal cohort analysis with participant fixed effects and repeated 24-hour dietary recalls before/after a combined national policy. Low- and middle-income neighbourhoods of southeastern Santiago, Chile; intake classified at school, home, and other locations. 349 FECHIC children aged 4–6 years at baseline, followed in 2016–2019; 294 GOCS adolescents aged 12–14 years, followed in 2016–2018; complete-wave analytic samples. | Chile's combined front-of-package warnings, child-directed marketing restrictions, and school sale/promotion restrictions for high-in products.  **Narrative contribution:** Dietary energy, total sugars, saturated fats, and sodium by eating location; no brand-response endpoint. | Children’s school-consumed energy share from sugars fell by 4.5 percentage points in 2018 (95% CI −8.0 to −.9) and 11.8 in 2019 (−15.4 to −8.3), relative to 2016. Adolescents’ school sugar and saturated-fat shares declined in 2018, while some nutrients increased at other locations. Longitudinal dietary analysis of Chile’s combined policy package; school and other policy components were jointly implemented. | T4 |
| FG-0453 Moran et al. (2017) Direct | Cross-sectional nutritional audit of products in a school-linked brand-reward program; product list July 2016, manufacturer data July–December 2016. United States; national Box Tops for Education program. 949 food/beverage products: 13 beverages, 171 entrees, 677 snacks and 88 exempt fruit/vegetable products. These are products, not schools or children. | Schools earn money through collection of box tops from participating branded products.  **Narrative contribution:** Product compliance with Smart Snacks nutrition criteria, assessed per serving. | Among Box Tops products, 69% of 13 beverages, 26% of 171 entrées and 23% of 677 snacks met Smart Snacks criteria. Overall, 24% of 848 nonexempt foods qualified. National program-product audit; per-serving nutrient classification measures product composition rather than consumption. | T1 T2 |
| FG-0463 Caspi et al. (2015) Direct | Repeated school-principal/designee surveys; adjusted generalized estimating equations across 2008, 2010 and 2012. Minnesota, USA; secondary schools across city, suburban and town/rural locations. 505 unique secondary schools; some repeated across years; annual principal response rates 70%–84%. | School bans on advertisements for candy, fast-food restaurants and soft drinks in buildings, grounds, transport and school publications/websites.  **Narrative contribution:** Adds longitudinal school-policy evidence showing that advertising restrictions can become less common. | City schools more often banned building ads than town/rural schools (adjusted difference 17.7 percentage points, 95% CI 5.5–29.9) and grounds ads (15.6, 1.7–29.5). Grounds-ad bans declined overall (p=.01); the city advantage in publication bans disappeared by 2012. Average differences by subsidized-meal eligibility or minority enrollment were absent. Repeated principal/designee reports from 505 unique secondary schools; 2008–2012. | T1 T2 |
| FG-0483 Paraje et al. (2026) Context | Cohort difference-in-differences analysis of administrative child anthropometry Chile; public and publicly subsidized schools; 2012–2017 321,597 children aged 4–6 years in four entry cohorts, followed through pre-kindergarten, kindergarten and first grade | National package combines warning labels, child-directed marketing restrictions and school sales restrictions  **Narrative contribution:** Adds a cohort difference-in-differences evaluation of the Chilean policy package and childhood excess weight. | At 18 months, estimated excess-weight probability fell by 2.85 percentage points for girls (95% CI −4.07 to −1.63) and 2.40 for boys (−3.58 to −1.22) relative to earlier cohorts. Six-month estimates were also negative; some subgroup estimates were nonsignificant. Combined-policy cohort difference-in-differences, 321,597 children; no contemporaneous untreated group. Eighteen-month pre-trends were untestable; girls’ BMI-for-age pre-trends marginally rejected parallelism. | T4 |
| FG-0499 Batty and Gee (2019), Sport Management Review, 22(1), 167–179 Context | Qualitative case study; semi-structured interviews and document analysis. New Zealand regional rugby organisations serving youth through adult grades. Six regional-organisation managers/CEOs; an additional district-health-board representative provided public-health context. Six manager interviews lasted 40–60 minutes. | Regional sponsorship involving fast-food and soft-drink brands; alcohol also considered.  **Narrative contribution:** Managerial accounts, sponsor relationships, and organisational finances; no child behavioural measurement or separately quantified youth-programme exposure. | Across six regional rugby organisations, all commercial sponsorship supplied 24–33% of annual revenue in 2015. Managers described funding shortfalls and uncertainty about potential sponsorship restrictions. Mixed-age regional sport governance, including youth; percentages cover all commercial sponsors, not food sponsors alone. | T2 |
| FG-0537 Shwed et al. (2023) Direct | Multiple instrumental qualitative case study; child/parent focus groups and coach interviews. Two recreational soccer leagues and two minor hockey associations in central/northeastern Ontario, Canada; 2019–2020. Main reported sample: 64 children aged 4–12, 30 parents, and 18 coaches (112 total). One Results sentence reports 20 coaches, differing from the 18 interviews and gender counts; Table 2 subgroup denominators also differ. | Fast-food sponsorship (including Tim Hortons/McDonald's) and coach/parent commercial-food rewards.  **Narrative contribution:** Adds children’s contrasting qualitative accounts of reward-related sport motivation. | Children enjoyed rewards but said they would still play without them; parents and coaches also did not view food as the main participation motivator. Sponsors were valued for containing fees. Participants identified no conscious sponsorship influence on treat choices or motivational methods. Qualitative perceived-motivation findings. Main sample: 64 children, 30 parents, 18 coaches; a 20-coach sentence and subgroup counts are inconsistent. | T2 T3 |
| FG-0552 Girona et al. (2019) Direct | Mixed-method school audit and principal interviews, July–November 2016. Montevideo, Uruguay, public/private primary and secondary schools. 59 schools: 38 primary (24 public, 14 private), 21 secondary (8 public, 13 private); principals interviewed. | Branded fridges/banners/displays, printed materials, company visits, games, gifts and informational talks.  **Narrative contribution:** Adds school implementation evidence from Uruguay concerning branded equipment and company activities. | Primary schools hosted company branding, games, gifts and talks. Point-of-sale advertising appeared in every school type except public primary schools. Compliance was low, with no secondary school compliant; principal awareness did not consistently reflect accurate knowledge of advertising restrictions. Audit and principal interviews in 59 schools; compliance concerns the assessed regulatory requirements. | T1 T2 |
| FG-0575 von Hippel and Bogolasky Fliman (2024) Context | Repeated annual population weight-surveillance comparison, 2013–2019; uncontrolled policy-package evaluation. Chile, government-funded schools; pre-kindergarten, kindergarten, first and ninth grade. Annual average 185,169 children per grade in 9,006 schools; average participation 85%. These are not unique longitudinal participants. | 2016 integrated warning-label, child-marketing and school-sales regulations.  **Narrative contribution:** Adds contrasting grade-level weight trends for the same Chilean policy package and surveillance system. | No sustained decline in overweight/obesity followed 2016 in any examined grade. Initial reductions in younger grades rebounded, while ninth-grade prevalence increased. Uncontrolled 2013–2019 repeated surveillance of Chile’s combined policy; annual mean 185,169 children per grade, not unique longitudinal participants. | T4 |
| FG-0716 Velazquez et al. (2015) Direct | Cross-sectional photographic audit of publicly accessible school common areas, November 2012–April 2013; purposive sampling. Vancouver, British Columbia, Canada; public elementary and secondary schools. 23 schools: 15 elementary and eight secondary; 493 food/beverage promotions recorded overall. | Food-product advertising and brand logos on school-controlled surfaces alongside student artwork and nutrition messages.  **Narrative contribution:** Promotion prevalence, location, purpose, brand/commercial characteristics and nutritional profile; no child intake or preference measurement. | Food promotions appeared in 20/23 schools (87%; median 17 per school). The audit recorded 493 promotions overall; 155/492 coded promotions (32%) were commercial. Approximately one-quarter of all promotions depicted foods prohibited for sale. Publicly accessible common-area audit; the overall denominator includes artwork and nutrition messages as well as commercial advertising. | T1 |
| FG-0717 Merlo et al. (2018) Direct | Cross-sectional linked state/district analysis of the 2012 School Health Policies and Practices Study. United States; state agencies and public-school districts. 51 state nutrition-services respondents; district nutrition-services n = 660 and general-school-environment n = 630. Marketing/fundraising models used 487, 480, 418 and 510 districts, respectively. | District restrictions on unhealthy-food advertising, distribution of branded products, soft-drink advertising and food fundraising.  **Narrative contribution:** District-reported restrictions and healthy-food promotion practices; no actual marketing exposure or student diet measured. | State guidance was associated with district advertising restrictions (AOR 1.54, 95% CI 1.14–2.07). Technical assistance was inversely associated with advertising bans (AOR .59, .46–.76) and branded-product distribution bans (.71, .55–.93); several other associations were nonsignificant. Cross-sectional state/district reports; outcome-model Ns were 487, 480, 418 and 510. Assistance may have targeted weaker policies. | T2 |
| FG-0761 Taillie et al. (2021), The Lancet Planetary Health, 5(8), e526–e533 Context | Before/after longitudinal household-purchase analysis; fixed-effects models compared post-policy purchases with a counterfactual extrapolated from pre-policy trends. Chile; Kantar WorldPanel households in cities with populations above 20,000, not a school-based child sample. 2,381 households, January 2015–December 2017; 67,890 household-month observations; median 35 months of follow-up. Two households with missing demographics were excluded. | Combined warning labels, child-directed marketing restrictions, and school/nursery sale and promotion bans for high-in products.  **Narrative contribution:** Nutrients and energy in recorded household packaged-food/beverage purchases per capita per day; not observed child consumption or purchases within schools. | Compared with extrapolated pre-policy trends, purchased energy fell by 16.4 kcal/capita/day (95% CI −27.3 to −5.6), sugar by 11.5 kcal (−14.6 to −8.4), saturated fat by 2.2 kcal (−3.8 to −.5), and sodium by 27.7 mg (−46.3 to −9.1). Increased not-high-in purchases partly offset high-in reductions. Household packaged-food purchasing under Chile’s combined policy package; selected product categories were excluded. | T4 |

# Source and sample notes

The current narrative uses 29 direct setting studies, 2 sport-sponsorship experiments, and 11 contextual publications. Study characteristics and findings are retained alongside theme codes and the specific contribution to the synthesis.

FG-0009, FG-0023, and FG-0016 report validation, baseline, and intervention components of the Eat Play Live facility framework. Facility sales concern purchasing, not child-specific intake. FG-0001, FG-0055, and FG-0058 share one national Canadian facility project, with analytic samples of 85 linked records, 86 manager surveys, and 134 photographic audits.

In FG-0058, 1,583 of 1,846 healthfulness-classifiable marketing instances were nutritionally of concern (85.8%); the abstract gives 86.5%. The wider photographic inventory contains 2,576 instances. Mixed food/beverage marketing totals in FG-0016 and FG-0001 may include alcohol; aggregate totals and food/non-alcohol-specific estimates remain distinct.

FG-0137 draws its 16 club-official interviews from the FG-0211 audit framework and also reports a separate parent survey. FG-0211 has 216 sport–club entries representing 191 distinct clubs. Minnesota ScOPE studies include overlapping schools or survey waves. Chilean evaluations FG-0483 and FG-0575 draw on the JUNAEB surveillance system with different linkage, comparison, and follow-up designs.

Reviews and policy inventories can reuse primary evidence or policy documents; their sample totals are not added to primary-study participant counts. Dietary interventions without commercial marketing in the broader Farmer et al. review are distinguished from its commercial-marketing content. Nutrient profiles of promoted products characterize exposure rather than measured dietary intake.

**Supplementary Table S2B. Historical and current narrative use of the original 41-publication candidate map.**

| **Record** | **Cited in original narrative** | **Cited in revised narrative** | **Archived candidate-map selection rationale** |
| --- | --- | --- | --- |
| FG-0011 Velazquez et al. (2017) | Yes | Yes | Foundational review of school food and beverage marketing evidence and measurement limitations |
| FG-0105 Carter et al. (2012) | Yes | Yes | Systematic review of food availability marketing sponsorship and stakeholder views in sport settings |
| FG-0049 Bragg et al. (2018) | Yes | Yes | Review connecting sport sponsorship athlete endorsement and youth food perceptions |
| FG-0106 Farmer et al. (2025) | Yes | Yes | 2025 scoping review of school marketing and sponsorship and food-choice evidence |
| FG-0113 Perry et al. (2024) | Yes | Yes | Global review of national policies restricting school food marketing and competitive food sales |
| FG-0003 Prowse (2017) | No | No | Settings-based review showing limited institutional-setting evidence and fragmented regulation |
| FG-0175 Probart et al. (2006) | No | No | Soft-drink advertising within high schools |
| FG-0201 Molnar et al. (2008) | No | No | Direct evidence on marketing foods of minimal nutritional value in schools |
| FG-0087 Polacsek et al. (2012) | No | Yes | Compliance with a statewide ban on junk-food and beverage marketing in schools |
| FG-0716 Velazquez et al. (2015) | Yes | Yes | In-school advertising messaging and signage audit |
| FG-0242 Larson et al. (2014) | No | Yes | School promotions trends and associations with dietary behaviors |
| FG-0453 Moran et al. (2017) | Yes | Yes | School brand-marketing program adherence to federal nutrition criteria |
| FG-0717 Merlo et al. (2018) | Yes | Yes | State guidance and district practices for school food marketing |
| FG-0030 Potvin Kent et al. (2019) | Yes | Yes | Canadian primary and secondary school marketing audit |
| FG-0061 Polacsek et al. (2019) | Yes | No | School-controlled digital marketing environments |
| FG-0093 Asada et al. (2020) | Yes | Yes | Superintendents' practices after national nutrition-policy change |
| FG-0299 Reeve et al. (2018) | Yes | Yes | Qualitative implementation lessons for school marketing restrictions |
| FG-0298 Tandoh et al. (2023) | Yes | Yes | Community readiness to address unhealthy marketing in and around schools |
| FG-0308 Fretes et al. (2023) | Yes | Yes | Dietary intake after an integrated law covering labeling advertising and sales in schools; attribution treated cautiously |
| FG-0761 Taillie et al. (2021) | Yes | Yes | Food purchases after integrated labeling marketing and school-sales policies; attribution treated cautiously |
| FG-0122 Kelly et al. (2011) | Yes | Yes | Who funds children's sport and what sponsorship relationships provide |
| FG-0173 Kelly et al. (2011) | Yes | Yes | Junior players' misconceptions and affective responses to food sponsors |
| FG-0080 Kelly, Bauman, and Baur (2014) | No | No | Population exposure to food and beverage sponsorship of sports clubs |
| FG-0143 Kelly et al. (2012) | No | No | Sporting-community attitudes toward sponsorship restrictions |
| FG-0023 Prowse et al. (2018b) | Yes | Yes | FoodMATS audit across policy environments |
| FG-0009 Prowse et al. (2018a) | Yes | Yes | Reliability and validity of a setting-wide marketing assessment tool |
| FG-0008 Dixon et al. (2018) | Yes | Yes | Experiment comparing unhealthy-food and pro-health junior-sport sponsorship |
| FG-0499 Batty and Gee (2019) | No | Yes | Regional sport organizations' sponsorship trade-offs |
| FG-0031 Pauzé et al. (2020) | Yes | Yes | Company sponsorship of children's sports clubs |
| FG-0002 Scully et al. (2020) | Yes | Yes | Parents' reactions to unhealthy-food and alternative sponsorship |
| FG-0159 Gonzalez et al. (2020) | Yes | Yes | Fast-food sponsorship in clubs with junior teams |
| FG-0069 Donaldson and Nicholson (2020) | No | No | Organization members' attitudes to junk-food sponsorship |
| FG-0016 Prowse et al. (2020) | Yes | Yes | Capacity-building intervention targeting facility marketing features |
| FG-0211 Martino et al. (2021) | Yes | Yes | State-wide audit of unhealthy sponsorship in junior clubs |
| FG-0086 Prowse et al. (2022) | No | No | Parents' interpretations of facility marketing and mixed health messages |
| FG-0137 Zorbas et al. (2023) | Yes | Yes | Perceptions of unhealthy sponsorship in junior sport |
| FG-0001 Prowse et al. (2025) | Yes | Yes | Marketing frequency by sponsorship agreements policies and food-service contracts |
| FG-0165 Pauzé et al. (2025) | No | No | Children's reported advertising exposure across two policy environments |
| FG-0055 Prowse et al. (2026) | Yes | Yes | Prevalence commercial value and methods of facility sponsorship and contracts |
| FG-0058 Lei et al. (2026) | Yes | Yes | Recent photo analysis of general and sport-related marketing techniques |
| FG-0089 Pauzé et al. (2025) | Yes | Yes | Disparities in sports participation and advertising exposure |

# References

FG-0093 Asada, Y., Harris, J. L., Mancini, S., Schwartz, M. B., & Chriqui, J. F. (2020). Food and beverage marketing in schools: School superintendents’ perspectives and practices after the Healthy, Hunger-Free Kids Act. *Public Health Nutrition*, *23*(11), 2024–2031. <https://doi.org/10.1017/s1368980019004804>

FG-0499 Batty, R. J., & Gee, S. (2019). Fast food, fizz, and funding: Balancing the scales of regional sport organisation sponsorship. *Sport Management Review*, *22*(1), 167–179. <https://doi.org/10.1016/j.smr.2018.06.014>

FG-0049 Bragg, M. A., Roberto, C. A., Harris, J. L., Brownell, K. D., & Elbel, B. (2018). Marketing food and beverages to youth through sports. *Journal of Adolescent Health*, *62*(1), 5–13. <https://doi.org/10.1016/j.jadohealth.2017.06.016>

FG-0105 Carter, M.-A., Edwards, R., Signal, L., & Hoek, J. (2012). Availability and marketing of food and beverages to children through sports settings: A systematic review. *Public Health Nutrition*, *15*(8), 1373–1379. <https://doi.org/10.1017/s136898001100320x>

FG-0463 Caspi, C. E., Davey, C., Nelson, T. F., Larson, N., Kubik, M. Y., Coombes, B., & Nanney, M. S. (2015). Disparities persist in nutrition policies and practices in Minnesota secondary schools. *Journal of the Academy of Nutrition and Dietetics, 115*(3), 419–425.e3. <https://doi.org/10.1016/j.jand.2014.08.029>

FG-0008 Dixon, H., Scully, M., Wakefield, M., Kelly, B., & Pettigrew, S. (2018). Community junior sport sponsorship: An online experiment assessing children's responses to unhealthy food v. pro-health sponsorship options. *Public Health Nutrition*, *21*(6), 1176–1185. <https://doi.org/10.1017/s1368980017003561>

FG-0106 Farmer, K. O., Beukes, R. A., Sowden, M., & du Plessis, L. M. (2025). The impact of food and nonalcoholic beverage sponsorship and marketing in the school environment on the food choices of school-going children and adolescents: A scoping review, 2011–2023. *Obesity Reviews*, *26*(8), e13923. <https://doi.org/10.1111/obr.13923>

FG-0308 Fretes, G., Corvalán, C., Reyes, M., Taillie, L. S., Economos, C. D., Wilson, N. L. W., & Cash, S. B. (2023). Changes in children’s and adolescents’ dietary intake after the implementation of Chile’s law of food labeling, advertising and sales in schools: A longitudinal study. *International Journal of Behavioral Nutrition and Physical Activity*, *20*(1), 40. <https://doi.org/10.1186/s12966-023-01445-x>

FG-0552 Girona, A., Iragola, V., Alcaire, F., Curutchet, M. R., Pereira, P., Magnani, D., Barreto, P., Petingi, S., Allegue, G., Conzalo, G., Mosteiro, C., & Ares, G. (2019). Factors underlying compliance with a healthy snacking initiative in the school environment: Accounts of school principals in Montevideo (Uruguay). *Public Health Nutrition*, *22*(4), 726–737. <https://doi.org/10.1017/s1368980018003488>

FG-0159 Gonzalez, S., Kingsland, M., Hall, A., Clinton-McHarg, T., Lecathelinais, C., Zukowski, N., Milner, S., Sherker, S., Rogers, B., Doran, C., Brooke, D., Wiggers, J., & Wolfenden, L. (2020). Alcohol and fast food sponsorship in sporting clubs with junior teams participating in the ‘Good Sports’ program: A cross-sectional study. *Australian and New Zealand Journal of Public Health*, *44*(2), 145–151. <https://doi.org/10.1111/1753-6405.12954>

FG-0122 Kelly, B., Baur, L. A., Bauman, A. E., King, L., Chapman, K., & Smith, B. J. (2011a). Food and drink sponsorship of children’s sport in Australia: Who pays? *Health Promotion International*, *26*(2), 188–195. <https://doi.org/10.1093/heapro/daq061>

FG-0173 Kelly, B., Baur, L. A., Bauman, A. E., King, L., Chapman, K., & Smith, B. J. (2011b). "Food company sponsors are kind, generous and cool": (Mis)conceptions of junior sports players. *International Journal of Behavioral Nutrition and Physical Activity*, *8*, 95. <https://doi.org/10.1186/1479-5868-8-95>

FG-0149 Kelly, C., Clerkin, P., Nic Gabhainn, S., & Mulvihill, M. (2010). Food marketing in Irish schools. *Health Education, 110*(5), 336–350. <https://doi.org/10.1108/09654281011068504>

FG-0242 Larson, N., Davey, C. S., Coombes, B., Caspi, C., Kubik, M. Y., & Nanney, M. S. (2014). Food and beverage promotions in Minnesota secondary schools: Secular changes, correlates, and associations with adolescents' dietary behaviors. *Journal of School Health*, *84*(12), 777–785. <https://doi.org/10.1111/josh.12209>

FG-0058 Lei, N., Warken, M., Michael, A., Benteau, T., & Prowse, R. J. L. (2026). General and sport-related marketing techniques in Canadian recreation and sport facilities: Cross-sectional photo analysis of food and beverage advertisements. *Public Health Nutrition*, *29*(1), e90. <https://doi.org/10.1017/s1368980026102377>

FG-0211 Martino, F., Chung, A., Potter, J., Heneghan, T., Chisholm, M., Riesenberg, D., Gupta, A., & Backholer, K. (2021). A state-wide audit of unhealthy sponsorship within junior sporting clubs in Victoria, Australia. *Public Health Nutrition*, *24*(12), 3797–3804. <https://doi.org/10.1017/s1368980021002159>

FG-0181 Marx, K., Greenthal, E., Ribakove, S., Grossman, E. R., Lucas, S., Ruffin, M., & Benjamin-Neelon, S. E. (2022). Marketing of sugar-sweetened beverages to youth through U.S. university pouring rights contracts. *Preventive Medicine Reports, 25*, Article 101688. <https://doi.org/10.1016/j.pmedr.2021.101688>

FG-0717 Merlo, C. L., Michael, S., Brener, N. D., & Blanck, H. (2018). State-level guidance and district-level policies and practices for food marketing in US school districts. *Preventing Chronic Disease*, *15*, 170352. <https://doi.org/10.5888/pcd15.170352>

FG-0453 Moran, A. J., Rimm, E. B., & Taveras, E. M. (2017). A school-based brand marketing program’s adherence to federal nutrition criteria. *American Journal of Preventive Medicine*, *53*(5), 710–713. <https://doi.org/10.1016/j.amepre.2017.06.017>

FG-0483 Paraje, G., Valdés, N., Vega Macaya, A., Corvalán, C., & Popkin, B. (2026). The impact of Chile’s multipronged food labelling and advertising law on early childhood excess weight: A cohort difference-in-differences study. *The Lancet, 407*(10548), 2630–2640. <https://doi.org/10.1016/S0140-6736(26)00651-3>

FG-0031 Pauzé, E., Ekeh, O., & Potvin Kent, M. (2020). The extent and nature of food and beverage company sponsorship of children’s sports clubs in Canada: A pilot study. *International Journal of Environmental Research and Public Health*, *17*(9), 3023. <https://doi.org/10.3390/ijerph17093023>

FG-0089 Pauzé, E., Roy-Gagnon, M.-H., Mah, C. L., Vanderlee, L., White, C. M., Hammond, D., & Potvin Kent, M. (2025). Disparities in children's sports participation and food advertising exposure in amateur sports settings in Canada. *Health Promotion International*, *40*(4), daaf107. <https://doi.org/10.1093/heapro/daaf107>

FG-0113 Perry, M., Mardin, K., Chamberlin, G., Busey, E. A., Taillie, L. S., Dillman Carpentier, F. R., & Popkin, B. M. (2024). National policies to limit food marketing and competitive food sales in schools: A global scoping review. *Advances in Nutrition*, *15*(8), 100254. <https://doi.org/10.1016/j.advnut.2024.100254>

FG-0087 Polacsek, M., O'Rourke, K., O'Brien, L., Blum, J. W., & Donahue, S. (2012). Examining compliance with a statewide law banning junk food and beverage marketing in Maine schools. *Public Health Reports*, *127*(2), 216–223. <https://doi.org/10.1177/003335491212700211>

FG-0030 Potvin Kent, M., Velazquez, C. E., Pauzé, E., Cheng-Boivin, O., & Berfeld, N. (2019). Food and beverage marketing in primary and secondary schools in Canada. *BMC Public Health*, *19*(1), 114. <https://doi.org/10.1186/s12889-019-6441-x>

FG-0009 Prowse, R. J. L., Naylor, P.-J., Olstad, D. L., Carson, V., Mâsse, L. C., Storey, K., Kirk, S. F. L., & Raine, K. D. (2018a). Reliability and validity of a novel tool to comprehensively assess food and beverage marketing in recreational sport settings. *International Journal of Behavioral Nutrition and Physical Activity*, *15*(1), 38. <https://doi.org/10.1186/s12966-018-0667-3>

FG-0023 Prowse, R. J. L., Naylor, P.-J., Olstad, D. L., Carson, V., Storey, K., Mâsse, L. C., Kirk, S. F. L., & Raine, K. D. (2018b). Food marketing in recreational sport settings in Canada: A cross-sectional audit in different policy environments using the Food and Beverage Marketing Assessment Tool for Settings (FoodMATS). *International Journal of Behavioral Nutrition and Physical Activity*, *15*(1), 39. <https://doi.org/10.1186/s12966-018-0673-5>

FG-0016 Prowse, R. J. L., Naylor, P.-J., Olstad, D. L., Storey, K., Carson, V., Mâsse, L. C., Kirk, S. F. L., & Raine, K. D. (2020). Impact of a capacity-building intervention on food marketing features in recreation facilities. *Journal of Nutrition Education and Behavior*, *52*(10), 935–943. <https://doi.org/10.1016/j.jneb.2020.03.009>

FG-0001 Prowse, R., Warken, M., Tran, T., Olstad, D. L., Kirk, S. F. L., Raine, K. D., & Hobin, E. (2025). Frequency of food marketing in recreation and sport facilities differs by presence of food sponsorship agreements and food service contracts in Canada. *Applied Physiology, Nutrition, and Metabolism*, *50*, 1–9. <https://doi.org/10.1139/apnm-2024-0517>

FG-0055 Prowse, R. J. L., Warken, M., Tran, T., Olstad, D. L., Kirk, S. F. L., Raine, K. D., & Hobin, E. (2026). Prevalence, value and food marketing methods of food-related sponsorship and contracts in Canadian recreation and sport facilities. *Public Health Nutrition*, *29*(1), e85. <https://doi.org/10.1017/s1368980025101699>

FG-0299 Reeve, E., Thow, A. M., Bell, C., Engelhardt, K., Gamolo-Naliponguit, E. C., Go, J. J., & Sacks, G. (2018). Implementation lessons for school food policies and marketing restrictions in the Philippines: A qualitative policy analysis. *Globalization and Health*, *14*(1), 8. <https://doi.org/10.1186/s12992-017-0320-y>

FG-0164 Rocha, L. L., Pessoa, M. C., Gratão, L. H. A., do Carmo, A. S., Cordeiro, N. G., Cunha, C. F., de Oliveira, T. R. P. R., & Mendes, L. L. (2021). Characteristics of the school food environment affect the consumption of sugar-sweetened beverages among adolescents. *Frontiers in Nutrition*, *8*, Article 742744. <https://doi.org/10.3389/fnut.2021.742744>

FG-0002 Scully, M., Wakefield, M., Pettigrew, S., Kelly, B., & Dixon, H. (2020). Parents' reactions to unhealthy food v. pro-health sponsorship options for children's sport: An experimental study. *Public Health Nutrition*, *23*(4), 727–737. <https://doi.org/10.1017/s1368980019003318>

FG-0537 Shwed, A., Bruner, B., Law, B., & Bruner, M. W. (2023). Investigating the impact of food rewards on children’s motivation to participate in sport. *Children, 10*(3), Article 432. <https://doi.org/10.3390/children10030432>

FG-0761 Taillie, L. S., Bercholz, M., Popkin, B., Reyes, M., Colchero, M. A., & Corvalán, C. (2021). Changes in food purchases after the Chilean policies on food labelling, marketing, and sales in schools: A before-and-after study. *The Lancet Planetary Health*, *5*(8), e526–e533. <https://doi.org/10.1016/s2542-5196(21)00172-8>

FG-0298 Tandoh, A., Laar, A., Pradeilles, R., Le Port, A., Osei-Kwasi, H., Amevinya, G. S., Aryeetey, R. N. O., Agyemang, C., & Holdsworth, M. (2023). Addressing the marketing and availability of unhealthy food and beverages in and around selected schools in Ghana: A community readiness appraisal. *BMJ Open*, *13*(9), e075166. <https://doi.org/10.1136/bmjopen-2023-075166>

FG-0256 Trübswasser, U., Talsma, E. F., Ekubay, S., Poelman, M. P., Holdsworth, M., Feskens, E. J. M., & Baye, K. (2022). Factors influencing adolescents' dietary behaviors in the school and home environment in Addis Ababa, Ethiopia. *Frontiers in Public Health*, *10*, Article 861463. <https://doi.org/10.3389/fpubh.2022.861463>

FG-0716 Velazquez, C. E., Black, J. L., & Ahmadi, N. (2015). Food and beverage promotions in Vancouver schools: A study of the prevalence and characteristics of in-school advertising, messaging, and signage. *Preventive Medicine Reports*, *2*, 757–764. <https://doi.org/10.1016/j.pmedr.2015.08.020>

FG-0011 Velazquez, C. E., Black, J. L., & Potvin Kent, M. (2017). Food and beverage marketing in schools: A review of the evidence. *International Journal of Environmental Research and Public Health*, *14*(9), 1054. <https://doi.org/10.3390/ijerph14091054>

FG-0575 von Hippel, P. T., & Bogolasky Fliman, F. (2024). Did child obesity decline after 2016 food regulations in Chile? *Revista Panamericana de Salud Pública, 48*, Article e16. <https://doi.org/10.26633/RPSP.2024.16>

FG-0056 Wilking, C., Moukalled, S., & Polacsek, M. (2023). Reducing student exposure to digital food and beverage marketing: Policy and practice recommendations. *Journal of School Health*, *93*(7), 638–643. <https://doi.org/10.1111/josh.13315>

FG-0137 Zorbas, C., Martino, F., Heneghan, T., Potter, J., Chisholm, M., Cameron, A. J., Sacks, G., Ananthapavan, J., & Backholer, K. (2023). Perceptions towards unhealthy food sponsorship in junior sports in Victoria, Australia. *Australian and New Zealand Journal of Public Health*, *47*(2), 100024. <https://doi.org/10.1016/j.anzjph.2023.100024>
